# The Role of Cells in Encoding and Storing Information: A Narrative Review of Cellular Memory

**DOI:** 10.7759/cureus.73063

**Published:** 2024-11-05

**Authors:** Ana I Flores, Mitchell B Liester

**Affiliations:** 1 Department of Psychology, University of California San Diego, San Diego, USA; 2 Department of Psychiatry, University of Colorado School of Medicine, Colorado Springs, USA

**Keywords:** cellular memory, dna memory, exosomes, intracardiac nervous system, non-neuronal memory, organ transplants, protein memory, rna memory, somatic memory, synaptic memory

## Abstract

Memory, a fundamental aspect of human cognition and consciousness, is multifaceted and extends beyond traditional conceptualizations of mental recall. This review article explores memory through various lenses, including brain-based, body-based, and cellular mechanisms. At its core, memory involves the encoding, storage, and retrieval of information. Advances in neuroscience reveal that synaptic changes and molecular modifications, particularly in the hippocampus, are crucial for memory consolidation. Additionally, body memory, or somatic memory, highlights how sensory experiences and traumatic events are stored and influence behavior, underscoring the role of implicit memory. Multiple studies have demonstrated that memories can be encoded and stored in cells. Evidence suggests that these memories can then be transferred between individuals through organ transplantation. Additionally, observations in organisms that lack a nervous system, such as bacteria, fungi, and plants, expand traditional memory concepts. This review highlights and compiles novel research from the last few decades that explores information encoding and storage at a cellular level across a wide variety of disciplines. Our aim is to integrate these findings into a cohesive framework that helps explain the role of cellular processes in memory retention and transfer. By compiling research across diverse fields, this review aims to establish a foundation for future investigation into the physiological and psychological significance of cellular memory. Despite substantial progress, critical gaps persist in our understanding of how cellular memory interfaces with neural memory systems and the precise pathways through which information is encoded, stored, retrieved, and transferred at the cellular level. There has been a noticeable lack of research focused on cellular memory, and more rigorous investigations are needed to uncover how cells participate in memory and the extent to which these processes influence human behavior and cognition.

## Introduction and background

A lifetime of information influences every decision, thought, and action we take. This framework of data is memory, and it allows us to recall past events to better understand our behavior in the present and to plan for the future. As humans, we hold a special attachment to our memories; they center on emotions, relationships, and personal growth. They give us a sense of continuity and coherence in our lives. In many ways, memory is the cornerstone of human cognition and consciousness. However, it is also important to understand that the formation of memories involves a variety of separate mechanisms, and the process of accessing memories is equally complex [1-3]. Despite the varied range of research that has improved our understanding of different kinds of memories and their mechanisms, many aspects of memory remain a mystery, creating a demand for advanced research to explore different pathways of information encoding.

At its very core, memory can be understood as the ability to encode, store, and retrieve information [4]. All kinds of data, from visual stimuli to fear responses, can be stored and read through encoding. In its simplest form, memory is encoded information, which is stored through mechanisms that include the firing rate of neurons and the enhanced strength of synaptic connections [5,6]. Research suggests that memory is encoded by activating specific neurons, which strengthens synaptic connections to create memory traces, allowing experiences to be stored and retrieved later [7].

However, increasing evidence demonstrates that synaptic memory does not explain all types of memory. For example, unicellular organisms, such as bacteria and slime molds, demonstrate the capacity for memory [8,9]. Additionally, multicellular organisms without a nervous system, such as plants, exhibit memory [10]. The human immune system possesses the ability to encode and store information regarding interactions with previously encountered pathogens without the use of synaptic memory. Evidence demonstrates that memory can be encoded and stored in cells throughout the human body using non-synaptic mechanisms. We refer to this extra-synaptic memory as “cellular memory.”

For the purposes of this review, we define cellular memory as the ability of individual cells to encode, store, and retrieve information. Cellular memory involves multiple unique locations within cells, and a variety of specialized processes are utilized to remember information. Cellular memory is distinguished from synaptic memory, which involves the encoding, storage, and retrieval of information via the strengthening of existing synapses or the formation of new synapses. A number of articles have reviewed synaptic memory [11-13], but no comprehensive review examining cellular memory could be found.

This review article includes an investigation into different types of cells that exhibit cellular memory, the locations within cells where information is encoded and stored, and an examination of the various mechanisms by which information is encoded and stored within cells. Evidence suggesting that cellular memories can be transferred from one individual to another via organ transplantation is examined, along with evidence describing cellular memory in unicellular and multicellular organisms that do not possess a nervous system. Additionally, the psychological implications of cellular memory are considered in the context of future research and applications. Finally, the implications of our findings regarding cellular memory are discussed, along with suggestions for future research.

## Review

Memory and learning

In terms of information processing, memory and learning are intertwined and can be understood to work in tandem to encode, store, and access information. To fully explore the possibility of cellular memory, establishing how memory is seen and validated by current science is essential. Learning is one of the most reliable and proven ways that memory is exhibited by living creatures, and we aim to establish that behaviors that exhibit learning or conditioning imply memory as a function, regardless of neuronal structure. Classical conditioning is a hallmark of memory and learning observed in many living organisms and is one of the most basic forms of memory and learning. Classical conditioning utilizes short-term memory and long-term memory in tandem to produce learning. The formation of a long-term association between a conditioned stimulus and an unconditioned stimulus relies on the active processing of information in short-term memory, with surprising stimuli requiring more rehearsal, which is facilitated by recent priming [14-17]. Extinction, which describes the fading of a conditioned response after the conditioned stimulus is presented alone, demonstrates that the original memory remains intact but is suppressed. The response can return if the unconditioned stimulus is reintroduced in the same context (reinstatement), suggesting that extinction is less a sign of forgetting but rather results from an inability to retrieve the information, which can be corrected. Animals perceive extinction as an exception tied to a specific environment; when the context shifts, the effects of extinction fade, and conditioned responses reappear. To effectively retrieve a learned response, the conditions during testing should be similar to those during learning [18]. Classical conditioning has also been shown to have peripheral effects on the physiology of the body outside of the brain; classical conditioning triggers changes in heart rate and blood pressure when a neutral stimulus is repeatedly paired with an aversive event, like a shock [19]. The body learns by anticipating and preparing for the outcome, adapting its responses before the event occurs, with the amygdala playing a key role in managing these cardiovascular changes. Eric Kandel’s Nobel Prize-winning research proved that classical conditioning is a basic form of memory that is present at a molecular level in many living organisms. 

Kandel investigated memory by using the giant marine snail *Aplysia*. He chose this animal due to the relatively small number of neurons in its brain (20,000 versus 1 trillion in mammals) and the large size of its neurons. Kandel and his colleague Irving Kupfermann identified a simple defensive reflex in the snail that involved the withdrawal of the gill upon stimulation of the siphon. When a weak tactile stimulus was applied to the siphon, both the siphon and gill were withdrawn into the mantle cavity for protection. Subsequently, Kandel found that this simple reflex could be modified by three different forms of learning: habituation, sensitization, and classical conditioning [2].

This discovery implied that learning must involve some form of memory storage, and indeed, it was found that each form of learning in *Aplysia* had two phases: a transient memory that lasted minutes and an enduring memory that lasted days. Subsequently, it was found that short-term memory does not require the synthesis of new proteins, whereas novel protein synthesis is required for long-term memory. This led to the discovery that short-term memory involves the release of neurotransmitters from presynaptic neurons, whereas long-term memory involves structural changes in synapses [2]. Kandel’s research, which expanded our understanding and definition of memory, provides the basis for further investigations into brain-based or synaptic memory. 

Brain-based/synaptic memory

During the 21st century, advancements in memory research have skyrocketed. This new research has contextualized memory as a form of information storage that involves a variety of chemical processes. This assumption forms the basis for research on cellular memory and beyond. With each developing study, the definition of memory has expanded and evolved to encompass a much broader range of possibilities [20]. Given the extensive reviews of synaptic-based memory and learning that exist [11,21,22], our aim here is not to reiterate those discussions in detail but rather to provide a concise overview to establish context for the current topic. Today, many contemporary neuroscientists believe the physical locus of memory is found in the synapses that connect neurons [22]. Neurons are specialized cells that use electrical and chemical signals to send information. Information is exchanged between different areas of the brain via neurons and can also be sent between the brain, the spinal cord, and the entire body. Synapses are the small spaces between two neurons that provide an opportunity for chemical communication, and they also include the neuronal membranes on both sides of this space [23]. Presynaptic neurons emit chemical messengers that alter the membrane potential of the postsynaptic neuron. If this signal is strong enough, the postsynaptic neuron generates an action potential that is communicated to a different neuron. There are estimated to be at least 1.25 trillion synapses in the human brain [24].

Short-term memory is hypothesized to involve changes in the strength of pre-existing synapses, whereas long-term memory is associated with the growth of new synapses [21]. Many studies support the view that memories are stored in the brain and that the hippocampus plays an integral role in memory storage and consolidation [25-27]. One model posits that the encoding of short-term memories occurs in the hippocampus, whereas long-term memories are stored in the neocortex [28]. Other models focus on specific neuronal structures, cellular mechanisms, chemical neurotransmitters, and more [29].

Comprehensive studies on the molecular, electrophysiological, genetic, and anatomical pathways of memory have highlighted the importance of molecular modifications at all levels of memory creation and storage, as well as the critical role they play in the long-term plasticity of the brain [1]. Long-term memory initially exists in a fragile state susceptible to various interferences but strengthens over time through consolidation and reconsolidation processes. These phases temporarily restore vulnerability upon retrieval, allowing for potential modification of memory traces. In simpler terms, weak memories can be made stronger, and strong memories can be weakened [1,30]. Additionally, in the dorsal hippocampus of the brain, proteins like CREB, C/EBPβ, and C/EBPδ play a key role in memory consolidation [1].

Research has also shown that emotionally charged events, both positive and negative, increase memory retention. Stress hormones, such as noradrenaline and glucocorticoids, modulate memory retention by regulating intracellular signaling pathways and controlling epigenetic modifications in the cells [1]. 

However, despite extensive evidence identifying the origin of memories in the cerebrum, growing evidence suggests that not all memories are stored in the brain. Consider the example offered by Professor John Lorber, a neurologist and professor of pediatrics at Sheffield University in England [31]. Professor Lorber evaluated a young student who had been referred by the campus physician at his university. The student had earned an honors degree in mathematics, had an IQ of 126, and was described as “socially completely normal.” However, when a CT scan was performed, Dr. Lorber found that “instead of the normal 4.5-centimeter thickness of brain tissue between the ventricles and the cortical surface, there was just a thin layer of mantle (cortex) measuring a millimeter or so” [31,32]. Instead of brain tissue, the student's cranium was filled with cerebrospinal fluid; however, despite a significant lack of synapses and neurons, the student was still able to learn and store memories.

Despite extensive verifiable evidence supporting the existence of synaptic, brain-based memory, a growing body of evidence suggests that memory may be stored in locations other than the cerebrum as well. Perhaps the connection between the mind and body is stronger than we have previously understood, and memories may involve body systems other than the brain. Increasing evidence suggests this is true and that numerous cells in the body possess the capacity to remember by encoding and storing information, indicating that synapses in the brain are only one location where memories are stored. While we strongly believe that synaptic, brain-based memory is the primary way that information is stored, external modes of information storage may serve as a “backup” drive of information for an organism’s survival. Cellular memory possibly works in tandem with the brain to store information about the environment, preferences, and dangers to support memory functions.

Body memory/somatic memory

An emerging field in psychology involves the exploration of body memory, which is the concept that multisensory information is remembered and stored in the body, and has profound effects on our identity and sense of self. The study of body memory does not disregard the important role of the brain in memory, but it does strongly suggest that the mind and body work together to store memories and experiences.

Also known as somatic memory, this theory explores how the body is capable of storing memories outside of the brain by utilizing implicit memory, an automatic and unconscious type of memory. Body memory consists of various components, including procedural, sensory, motor, emotional, situational, intercorporeal, incorporative, pain, and traumatic memory [33]. It plays a significant role in trauma, where memories are stored with heightened intensity, driving symptoms through physiological arousal and reactivated sensations. The body, in this framework, actively draws on these stored experiences to interpret and respond to its surroundings [34]. Our subjective experiences and self-development are shaped by the integration of these sensory inputs into the body matrix, which predicts and adjusts to incoming sensory information to minimize errors and dynamically guide actions based on these predictions [35]. Other researchers have proposed a sensorimotor model of memory (SMM), in which bodily actions simulate sensory experiences of remembered events, indicating that manipulating the body can influence memory processes [36]. When recalling memories, evidence suggests that the details of body posture, movement, and actions experienced during encoding are reactivated [36,37].

Perhaps one of the most well-known examples of body memory is traumatic memory: the encoding of past traumatic life events, resulting in persistent memory traces in the body, even in the absence of conscious memory [38]. Traumatic memory has been shown to result in physical manifestations, such as pain, even years after the traumatic event occurred. Following stressful situations, patients suffering from posttraumatic stress disorder (PTSD) often experience pain or panic in response to stimuli that remind them of the original traumatic event. This body memory produces somatic sensations in response to the stimuli, suggesting that the body itself is “remembering” the event, and these trauma memories are stored and relived through the sensory modalities by which they were originally experienced [39,40]. Further research shows that emotions may be key to the recall of these body memories via up-regulation of the amygdala and down-regulation of the hippocampus [41]. Body memory has become the focus of many new treatment approaches to trauma and PTSD, which focus on the body’s role in healing and psychological well-being. Techniques like biofeedback, body illusions, and movement therapies improve bodily awareness and reduce symptoms by re-training responses to internal signals [34]. 

Phantom limb pain and studies on proprioceptive memory emphasize the deep connection between our bodies and brain, and the subconscious realm that stores those memories. Researchers investigating phantom pain in amputees theorized that the pain originated in the remains of proprioceptive memory, a body memory stored in the subconscious that records the position of our limbs in space and in relation to ourselves [42,43]. Supported by studies in patients undergoing anesthesia, paralyzed patients, and amputees, the theory posits that the brain stores proprioceptive memory subconsciously, and phantom limb sensations arise from the memory of the last input from the body before injury. Furthermore, a network of pain memories exists that is associated with each limb and serves as a defense mechanism, and during the traumatic process of amputation, signals from pain receptors in the limbs are stored in proprioceptive memory [42].

One distinguishing characteristic of somatic memory, as compared with synaptic memory, is that the former encodes and stores information in the body’s cells [44-46], whereas the latter encodes and stores information in synapses in the brain [21]. Numerous intracellular sites and mechanisms are employed by cells to remember and adapt to changing environmental conditions. Additional evidence supporting the existence of memory storage outside of the brain comes from research into personality changes following organ transplantation. This research has found that individuals who undergo organ transplantation can acquire the personality characteristics of their donor, suggesting that memories may be stored in these donated organs [47,48].

Cellular memory

For survival and evolutionary purposes, memory is essential if we are to evade danger, avoid repeating mistakes, or circumvent adverse consequences [49]. Additionally, our body and brain store a plethora of information that determines every little detail of ourselves, from preferences to personality [50]. Cellular memory, also known as somatic memory, describes the ability of cells to remember.

All living organisms are made up of cells. Unicellular organisms, such as bacteria and protozoa, have just one cell, whereas multicellular organisms, such as plants and animals, have multiple cells. A cell, which is the smallest unit of life capable of performing life functions, contains chromosomes made of DNA and proteins in the nucleus, along with multiple types of RNA. Each of these subcellular components possesses the capacity to encode and store information, and thus form memories.

In theory, all of the body’s cells possess the capacity to remember. Multiple parts of the cell are theorized to store information, including cell membranes [51], DNA [52], RNA [53], and proteins [54]. Examples of specific types of cells that exhibit cellular memory include immune cells [44] and skeletal muscle cells [45,55].

Immunological memory

Immunological memory, a function of the adaptive immune response, describes the immune system’s ability to recognize and rapidly respond to previously encountered pathogens [44]. Once a pathogen enters a host, it is recognized as foreign or “non-self” by a group of immune cells known as “antigen-presenting cells” (APCs). These APCs engulf the pathogen and digest it, then express antigens from the digested pathogen on their surface. These antigens are then presented to T cells, resulting in T cell activation, differentiation, and expansion. After the pathogen is cleared, most of the effector T cells die, but some survive and become long-lived memory T cells. This immune response produces a reservoir of long-lived antigen-specific cells that remember the pathogen and can respond more quickly and more robustly to subsequent encounters with the same pathogen [56,57]. 

Several types of immune cells retain a memory of previously encountered pathogens, including memory T cells, memory B cells, and natural killer cells. These cells circulate in the blood and persist in the spleen and lymph nodes, where they monitor for the specific antigen they “remember” [58]. When memory T cells re-encounter a previously encountered antigen, they generate a more rapid and effective immune response than naive T cells are capable of producing. Memory B cells also undergo a process known as “affinity maturation,” which can be thought of as a type of enhanced memory in which these cells acquire increased specificity and affinity for pathogens, resulting in a more robust and effective immune response [59]. During the first encounter with a novel pathogen, it can take two weeks to generate enough antibodies for them to be detected. However, following affinity maturation, antibodies can be detected within two to four days of exposure [60].

Neuromuscular memory

Another type of cellular memory is neuromuscular memory. Skeletal muscles, which account for up to 40% of the total body mass, consist of contractile units known as multinucleated myofibers, which are constructed of large cells containing multiple nuclei and a contractile apparatus made of actin and myosin proteins. Myofibers vary in size, metabolic capacity, mitochondrial content, and contractile properties. During fetal development, specialized myofiber phenotypes are programmed, and these are later modulated in response to changing motor nerve input. This plasticity allows myofibers to alter their cellular capacities in response to varying physiological demands. These adaptive changes are minimal following a single episode of neuromuscular activity. However, they manifest fully after repeated bouts of physical activity occurring over a period of days to weeks, as occurs during training in human athletes. Muscle plasticity thus involves a form of cellular memory [45,55]. 

Skeletal muscle also exhibits memory at the level of neuromuscular junctions (NMJs). NMJs are specialized connections between the terminal end of a motor nerve and a muscle fiber. These connections allow electrical impulses to be transmitted from the nerve to the muscle via chemical messages, resulting in contraction of the muscle. When skeletal muscle is injured, denervation may occur, resulting in the loss of NMJs. Reinnervation is crucial for the muscle to regenerate, a process that restores muscle tissue, nerves, and NMJs. Following injury, skeletal muscle guides the axon to form new NMJs at the same location where they existed in the pre-injured muscle. This restoration of NMJs at the site of previous NMJs demonstrates that muscles retain a memory of the location of NMJs [55].

DNA memory

In addition to the immune system, another location where information is encoded, stored, and retrieved is in DNA. At least four different mechanisms exist for encoding information in DNA: genetic memory, non-coding DNA memory, epigenetic memory, and electromagnetic (EM) energy.

One method for encoding and storing information in DNA is in the form of genes. The set of all codons is called the genetic code, and information encoded and stored in DNA is called genetic memory [52]. Genetic memory thus involves information encoded in a series of nucleotide bases, which constitute the “language” of genetic memory.

Large amounts of information can be stored utilizing DNA, as researchers at the European Bioinformatics Institute demonstrated. These scientists selected five computer files to encode, and they chose a range of common formats. The five files included all 154 of Shakespeare’s sonnets (ASCII text), a copy of Watson and Crick’s 1953 paper describing the molecular structure of DNA (PDF format), a color photograph of the European Bioinformatics Institute (JPEG 2000 format), a 26-second excerpt from Martin Luther King’s 1963 “I Have a Dream” speech (MP3 format), along with the Huffman code that was used to convert bytes to base-3 digits (ASCII text). This totaled 757,051 bytes of information. The computer files were then encoded into strings of synthetic DNA that were synthesized using an updated version of Agilent Technologies’ OLS (oligo library synthesis) process. Each DNA sequence was split into overlapping segments, producing fourfold redundancy. The synthesized DNA was then lyophilized and shipped without special packaging from the USA to Germany. The DNA strings were then reconstructed in silico. Those containing uncertainties due to synthesis or sequencing errors were discarded. The remaining strings were decoded using the reverse of the encoding procedure. Full-length DNA sequences representing the original encoded files were then reconstructed in silico, and the original computer files were reconstructed with 100% accuracy [61]. 

About 1% of our DNA codes for proteins, while the other 99% is noncoding DNA, i.e., it does not code for proteins. Noncoding DNA contains sequences that determine when and where genes are turned on and off. These regulatory elements are sites where transcription factors attach to DNA and turn on or off the processes by which genetic information is converted into proteins. Certain regions of noncoding DNA are involved in RNA processing and regulate the conversion of messenger RNA (mRNA) into proteins. These include transfer RNAs (tRNAs), ribosomal RNAs (rRNAs), small nuclear RNAs (snRNAs), small nucleolar RNAs (snoRNAs), and others [62].

A third method for encoding and storing information utilizing DNA is epigenetic memory. Rather than encoding information in a series of nucleotides on a strand of DNA, information is encoded in the way DNA is packaged and modified [63]. Epigenetic changes occur when enzymes attach or remove molecules to or from chromatin, or when RNAs are produced, resulting in a modification of gene expression. Examples of epigenetic changes include DNA methylation, histone modification, and the production of microRNAs (miRNAs). This entire process can either enhance or suppress the production of gene products [46]. Persisting epigenetic changes create an epigenetic code that determines whether a specific gene is transcribed [64]. Epigenetic changes also encode information that can be stored and retrieved over time. The entirety of an individual’s epigenetic changes at any given point in time is known as the “epigenome” [65].

The information saved in the epigenome creates a historical record of interactions between an individual and their environment. Stored as chemical and structural alterations of chromatin or short strands of RNA, this information persists as a type of cellular memory known as “epigenetic memory.” The epigenome provides a mechanism for encoding, storing, and retrieving interactions between the environment and an individual’s genome [63]. Epigenetic changes are stored in cells in a way that facilitates rapid adaptation to environmental changes - a process known as Lamarckian evolution [66].

Epigenetic memory can also be passed down from one generation to the next. This is known as “transgenerational epigenetic inheritance” or “epigenetic inheritance” [67,68]. Epigenetic inheritance allows for a person’s health or development to be influenced by the lived experiences of not only their parents but also their grandparents and previous generations, transferred through epigenetic memory embedded in genetic material. A recent study showed that the transmission of epigenetic memory can occur across multiple generations. By manipulating a histone modification (H3K27me3) in sperm chromosomes, which is typically involved in gene repression, researchers observed altered gene expression in offspring, suggesting a direct transmission of epigenetic memory [69]. Another study investigated pregnant women who were present in New York City during the World Trade Center attacks on September 11, 2000. Researchers found that their offspring carried epigenetic signs of PTSD, an indication of the trauma their mothers had experienced. Mothers who had PTSD gave birth to babies with lower cortisol levels, an epigenetic change that occurred in the womb [70-72].

A fourth mechanism by which information can be stored in DNA is via EM memory. During transcription, when DNA is converted to RNA, the two polynucleotide strands of DNA are separated, and the hydrogen bonds are broken. As a result of the breaking of these hydrogen bonds, a modulation occurs in the magnetic field in the form of an EM signal. This EM signal can send information from the cell nucleus to other cells. At the receiving cells, this process is reversed, and the transported information is converted from a magnetic wave back into a chemical structure [73]. Researchers have suggested that the ability to emit EM waves may be a property of all double-stranded helical DNAs [74,75]. Montagnier et al. found that treatment of DNA with DNase, which degrades DNA, destroys the DNA’s ability to emit EM signals. This demonstrates that it is the DNA and not some other subcellular component that emits the EM signals [75].

RNA memory

Similar to DNA, RNA contains information that is encoded, stored, and can be decoded in order to retrieve that information. Investigations exploring whether RNA-based memory can be transferred between different organisms have produced mixed results. One study extracted RNA from the brains of rats that had been trained to approach a food cup in response to a sound. When this RNA was injected intraperitoneally into untrained rats, no change in their behavior was observed [76].

However, research conducted at UCLA demonstrated that information stored in RNAs can be transferred from one animal to another. This study found that large sea slugs, known as *Aplysia*, could be trained to retract their siphon (a tube-like structure used to draw in water) by applying a mild electrical shock to their tail. RNA was then extracted from these trained *Aplysia* and injected into untrained *Aplysia*. The untrained sea slugs responded to the electric shocks as if they had been previously trained, withdrawing their siphons for a much longer period of time than control slugs. This enhanced withdrawal response was blocked if DNA methylation was inhibited via injection of the DNA methylation inhibitor RG-108. This suggests that a memory of the electrical shock may have been stored epigenetically in the DNA, and this memory may have been subsequently transferred via the RNA of the trained *Aplysia* to the untrained *Aplysia* [53].

Numerous mechanisms are utilized to encode and store information in RNA. One method involves transferring information that is stored in the genetic sequence of the DNA code into an RNA nucleotide sequence and then utilizing that information to produce proteins. This is referred to as transcription, and the type of RNA that is involved in this process is known as mRNA. RNAs that do not code for proteins are known as noncoding RNAs (ncRNAs).

Kim and Eberwine [77] have proposed that the RNA “transcriptome,” which is the sum of all the mRNAs expressed by an organism, represents a “snapshot memory” of cellular signals that regulate and maintain cellular phenotypes. They describe how alterations in the transcriptome can alter a cell’s phenotype from one differentiated cell type to a different differentiated cell type. Thus, manipulation of the transcriptome may induce reprogramming and reorganization of cellular components, including proteins, chromatin, and others. The transcriptome is viewed as “a set of tunable variables” that can be manipulated to create a desired cellular phenotype. It would seem plausible that, in order to create a new phenotype, a memory of the desired phenotype could be stored within the transcriptome.

Crisp et al. [78] have described how adaptation to environmental changes influences the composition of the transcriptome. Two factors affecting the composition of the transcriptome are the rates of transcription and mRNA decay. The total array of mRNA found in a cell at any given point in time is determined by the rates of transcription and degradation. Environmental alterations can induce a shift in the rates of transcription and degradation, thereby altering the steady state of the transcriptome and, thus, the information stored within it. This encoded information creates a type of transcriptome memory regarding interactions between an individual and its environment. This encoded information establishes a form of memory, highlighting how RNA not only stores data about environmental conditions but also guides cellular behavior based on these historical interactions.

Post-transcriptional modifications of RNA are essential for the function of various RNA types and can be categorized into RNA editing and RNA modifications. RNA editing involves changes to the nucleotide sequence through insertions, deletions, or deamination, while RNA modifications involve alterations in the chemical composition or conformation of a nucleotide, which can influence the RNA transcript's function or stability. Together, these processes regulate gene expression at the post-transcriptional level and allow cells to retain information by encoding it directly into the RNA sequence and utilizing it to influence future cellular behavior [79].

Interactions between an organism and its environment can result in RNA modifications. More than 150 different modifications of coding and non-coding RNAs have been reported thus far [80]. The sum of the modifications to RNA within a cell that do not alter the RNA's nucleotide sequence is known as the “epitranscriptome.” Similar to epigenetic changes in DNA, the transcriptome and the epitranscriptome contain encoded and stored information that forms a memory of previous interactions between an organism and its environment [79].

In summary, RNA influences memory via multiple mechanisms. These include the direct encoding and storage of information in the RNA nucleotide sequence, which is translated to form proteins; alterations in the transcriptome and epitranscriptome that create a memory of interactions between an organism and its environment; and initial evidence suggesting that it may be possible to transfer memories between individuals via the exchange of RNA.

Protein memory

Just as the packaging of DNA into chromosomes results in epigenetic memory, the folding of proteins can also influence memory. One type of protein that influences memory is a prion. Prions are proteins that can shift between and exist stably in multiple functionally distinct conformations, and at least one of these conformations is self-replicating [54].

Prions were discovered in the 1980s by Stanley Pruisner, who found that they cause transmissible, fatal neurodegenerative diseases such as Creutzfeldt-Jakob disease, bovine spongiform encephalopathy (Mad Cow disease), and kuru [81,82]. In addition to causing disease, prions are also a channel for the replication of heritable information, similar to DNA or RNA, and assist with the formation of long-term memory in humans [54,83-85]. The replication of prions provides a durable form of molecular memory [54].

In plants, prion-like proteins have been found that encode stress memory, which occurs via priming. Priming occurs when a previous brief exposure to stress primes the plant for future episodes of stress by facilitating a more rapid and heightened response of resistance. Prion-like proteins have been associated with a wide range of stress and memory processes, including flowering time and thermosensory responsiveness [83]. Studies in mice have uncovered a physiological function for the cellular prion protein that causes prion diseases. Mice bred without this gene were found to have impaired long-term learning and impaired long-term memory [84].

Researchers have also discovered a prion-like protein, polyadenylation element binding protein (CPEB), in the sea slug *Aplysia californica* that helps with memory by contributing to the maintenance of long-term changes in synapses. CPEB stimulates the translation of the mRNAs it regulates, leading to the production of proteins essential for maintaining long-term synaptic changes associated with memory storage [86]. As new synapses form during learning, soluble prions in these synapses convert into aggregated forms, which then trigger the synthesis of additional proteins necessary for memory maintenance. Long-term memory persists as long as these aggregated proteins are present, continuously recruiting newly synthesized soluble prions into their structure [87]. Additionally, single-nucleotide polymorphisms (SNPs) of the prion protein gene (PRNP) are associated with alterations in long-term memory in healthy humans. Researchers found that a specific SNP has been shown to improve long-term memory by 17% without affecting short-term memory [85]. In this way, prions serve as a mechanism for aiding and enhancing synaptic memory through mechanisms outside of the brain. Their relationship with memory, as well as their ability to encode information, could be an interesting area of research to study different pathways supporting the storage of memory in neurons.

Microtubules

Another type of cellular memory involves hollow, tube-like structures in cells known as microtubules. Microtubules are a type of cytoskeletal protein found in all living cells. These structures are polymers composed of repeating subunits of tubulin that provide shape and structure to cells, including neurons. Changes in microtubules have been postulated to play an important role in memory [24,88].

Microtubule-associated proteins (MAPs) are proteins that bind to microtubules in cells, where they regulate the assembly and stability of microtubules, as well as performing other important functions, such as information processing [24,89].

MAP2 is a microtubular protein that is found in high concentrations in neurons. MAP2 is particularly abundant in dendrites, where it serves as a signal-transduction molecule and is involved in memory acquisition and consolidation. The role of MAP2 in memory involves this protein's ability to influence neuroplasticity. MAP2 contributes to spine formation and maintenance, which is necessary for the acquisition and consolidation of memory. Disruption of MAP2 can disrupt the structural and functional plasticity of synapses, resulting in memory impairment [24,90].

Furthermore, microtubules undergo a process of polymerization and depolymerization known as “dynamic instability.” Microtubule dynamics is an ongoing process in all cell types, and recent studies have suggested that they play a role in memory and may be compromised in degenerative diseases like Alzheimer’s [88].

Although current studies suggest microtubules play a role in neuronal memory, information on this subject remains sparse, and further studies are needed to elucidate the role played by microtubules in memory.

Cell membranes

Cellular biologist and former Stanford researcher Bruce Lipton has demonstrated that the cell membrane acts as a site for encoding and storing information. The membrane is composed of a phospholipid bilayer embedded with integral membrane proteins (IMPs), which perform essential functions such as transporting molecules across the membrane. These IMPs also play a critical role in transmitting information between the cell's internal and external environments, allowing the cell to respond to external signals and transfer information [51]. 

IMPs can be divided into two functional classes: receptor proteins and effector proteins. Receptors function like antennae, which are tuned to receive information from specific environmental signals. Different receptor proteins respond to different environmental signals. Some receptor proteins even respond to vibrational energy fields, such as light, sound, and radio frequencies. If the energy frequency in the environment resonates with the frequency of the receptor, the protein’s charge is altered, resulting in a change in the protein’s shape and function [51].

The cell membrane is also capable of remembering. A study by Yang et al. [91] demonstrated that even bacteria are capable of storing information in their cell membranes. They accomplish this by modifying membrane proteins in a way that alters the cell’s response to subsequent external inputs.

Research by Condray et al. [92] suggests that cell membrane fatty acids are associated with semantic memory in schizophrenia. Abnormal fatty acid composition has been identified in the frontal cortex of individuals with schizophrenia, and these individuals exhibit impairments in semantic memory.

Evidence for cellular memory (organ transplant recipients)

We have now presented numerous examples of how and where memory can be encoded, stored, and retrieved in the body outside the brain. But does evidence exist to support the existence of cellular memory?

Neuropsychologist Paul Pearsall explored personality changes in heart transplant recipients and found that these individuals can take on the personality characteristics of their donor, despite having no information about their donor prior to their transplant [47]. Subsequent research found that changes in the personalities of heart transplant recipients mimicked the personality traits of their donors. These changes included preferences for food, music, art, sex, recreation, and career, as well as transplant recipients recalling the names and sensory experiences of their donors. This suggests that memories can be transferred from donor to recipient via the transplanted heart [48].

Claire Sylvia, who was the first person to receive a heart-lung transplant at the Yale University School of Medicine, documented the changes she experienced after her transplant, including developing an intense craving for chicken nuggets and green peppers - foods she previously disliked. Only later did she learn these were her donor’s favorite foods [93]. Sylvia is one of many heart transplant recipients who have reported changes in their interests, personality, likes and dislikes, and attitudes following transplant surgery. However, these reports remain largely uninvestigated due to the immediate dismissal of such accounts by healthcare providers, which stigmatizes transplant recipients and discourages them from reporting their experiences.

Despite numerous challenges, research has begun to investigate the prevalence of personality changes in organ recipients. A cross-sectional study found that up to 89% of all transplant patients experienced self-reported personality changes following organ transplantation, regardless of which organ was received. These changes were equally likely to occur whether the transplanted organ was a heart or a different organ [94]. Additionally, it is important to note that these personality changes include a wide range of experiences, from newfound preferences for food and music to changes in temperament and behavior. This wide range of changes makes it difficult to pinpoint the exact role of cellular memory and the types of information that may be encoded and transferred with the transplanted organ. Various studies across the globe have found that organ recipients struggle to integrate their sense of “self” and “other” following transplantation. Many also attribute newly acquired characteristics or traits to the organ they received. However, deeper investigations into these accounts rarely occur [95,96].

Further research is needed to fully understand the etiology of these personality changes. Even if cellular memory is not responsible for these changes in personality, a larger effort from the psychological community is needed to investigate the impact of these experiences on organ transplant recipients. Dismissal, lack of consideration, and outright refusal to listen to patients’ experiences cause distress for those experiencing these changes, as well as fear for those waiting for an organ transplant. Additional research on the role of cellular memory in organ transplants may be key to improving transplant recovery and the emotional well-being of recipients following such an emotionally charged surgery.

Cellular memory and the heart

Another question that remains to be answered is: how can memories be transferred from a donor’s heart to a recipient? Cardiac cells contain all of the cellular components that have previously been identified with memory. These include DNA, RNA, prions, microtubules, EM waves, and cell membranes. If memories are stored in these cellular components, it might be possible for the recipient of a new heart to retrieve these memories from the donor’s heart. Furthermore, research suggests tiny vesicles known as exosomes present a potential mechanism for transferring memories stored in the heart to cells in other organs of the body [97]. Exosomes play a vital role in intercellular communication by transferring proteins and various types of nucleic acids, such as DNA and RNA, from one cell to another [98-100]. Exosomes can cross the blood-brain barrier (BBB) [101], and this passage occurs bidirectionally [102]. Thus, memories from the donor’s life could be encoded in nucleic acids or proteins originating in the heart of the donor, and these memories could be transferred to the recipient’s brain following transplantation. Exosomes originating in the transplanted heart could then carry information about the donor, including memories, to the recipient’s brain, where the information could be decoded and retrieved.

Exosomes could transport information from other organs to the brain as well. Researchers at the University of Geneva found that blood transfusion recipients described changes in their mood, behavior, and memories after receiving blood from another person [103]. These findings suggest that blood transfusions may result in personality changes, and a potential mechanism explaining these changes is the transfer of information via exosomes from donor organs to recipients.

In addition, the heart possesses its own intrinsic nervous system known as the “intracardiac nervous system” [104] or the “heart-brain” [105]. This system includes an intricate network of neurons, neurotransmitters, proteins, and immune cells, all of which are capable of encoding, storing, and retrieving information. Studies have shown that the neural network of the heart is involved in decision-making and storing both short-term and long-term memories, with approximately 40,000 sensory neurites potentially playing a critical role in memory transfer [106]. These specialized nerves form connections with the recipient's body following the transplantation of the organ. Additionally, electrochemical signals originating from the “heart-brain” are delivered via afferent neurons in the spine and the vagus nerve, and are directed to several key brain regions, such as the medulla, hypothalamus, thalamus, and amygdala, suggesting a potential role in originating and transmitting memory [107]. If intracardiac neurons contain memories, it is possible that their interweaving with the recipient's nervous system following transplantation could grant access to donor memories.

Further evidence for cellular memory/non-neuronal memory

Living organisms without a traditional centralized nervous system or altered methods of neural tracts provide further evidence for alternative memory encoding and storage. Bacteria have multiple ways of remembering information, including genetic, heritable memory involving the inversion of specific DNA sequences, epigenetic changes [8], and autocatalytic loops whereby proteins establish the conditions necessary for their continued synthesis [108].

Slime molds, a group of unrelated eukaryotic organisms without brains, exhibit behaviors that require memory, such as moving toward food and solving mazes [109]. One example is the slime mold *Physarum polycephalum*, which is a large amoeba-like cell that changes shape as it moves. This single-celled organism is also capable of solving a labyrinthian maze by first exploring all possible paths, then changing its shape to form a single thick tube covering the shortest distance between the two food sources. This suggests that the slime mold can remember which paths provide the shortest distance to the desired goal [9].

Fungi have demonstrated the capacity to remember, even though they have no brain, central nervous system, or neural network. In one study, researchers primed a group of grassland fungi with high temperatures and then exposed them to a severe heat shock. In comparison to a control group, the primed fungi grew more when exposed to the second shock, suggesting that the compensatory mechanisms from the earlier high temperatures were remembered and deployed quickly at the second shock. These primed fungi also remembered the first stressful shock for up to 12 hours after the event, indicating prolonged memory storage despite a lack of a neural network [110]. According to mycologist Nicholas P. Money, mycelia exhibit spatial recognition, decision-making, learning, and short-term memory, and may even possess the capability of consciousness [111].

A fascinating insight into the storage of memory outside the brain has been demonstrated in planarian flatworms, a non-parasitic flatworm that can regenerate any part of its body through its abundance of adult pluripotent cells. Despite their small size, planarian flatworms have a centralized nervous system, a brain, and can regrow a new head after it is severed from its tail. Researchers trained planarian flatworms using classical conditioning and then cut the planarian flatworms in half. The flatworms that regenerated from the tail half formed an entirely new head and brain. These previously headless flatworms were found to retain memory of their conditioned learning [112,113]. This strongly suggests that the physical body stored memory that was transferred to the new brain after regeneration, supporting the existence of cellular memory.

In a recent study designed to explore the extent of memory encoding in planarian regeneration, researchers tested planarians' aversion to light by placing them in environments with different textures - either a ridged surface or a smooth Petri dish. They then illuminated a portion of the environment that contained food so the planarians would learn to overcome photoaversion. Planarians on the ridged surface overcame their aversion faster than those on the Petri dish. After decapitating and allowing the heads to regrow, all planarians retained some aversion to light, but those originally on the ridged surface showed quicker adaptation upon re-exposure, indicating lasting environmental familiarity and memory retrieval capabilities. Furthermore, this memory persisted for at least 14 days post-regeneration [114]. While the specific mechanisms for memory storage in planarian tissue still remain a mystery, these flatworms have become a key focal point in the investigation of memory encoding in biological tissue.

The complex mystery of memory formation and storage continues in the exploration of memory in animals that undergo metamorphosis. During metamorphosis, animals such as butterflies or moths undergo a process in which a caterpillar completely dissolves its body, leaving behind imaginal discs that become the basis of the new body structure. Inside the chrysalis, the protein-rich soup of dissolved tissue is rebuilt into the body of an adult butterfly. Throughout metamorphosis, insect brains undergo substantial remodeling: new neurons are incorporated while existing larval neurons are restructured or eliminated [115]. As larvae undergo metamorphosis to become adults, only some of their neural compartments are integrated into the adult mushroom body. Within these compartments, neurons either die off or undergo transformations to serve new adult functions. All connections between neurons in the mushroom body and their input and output neurons are dissolved during this developmental transition [116]. However, despite this complete reorganization of all existing synapses and neurons, adult insects have been shown to retain memories formed before metamorphosis. Researchers found that a species of moths, Lepidoptera, retained odor aversions learned in larval stages, suggesting that associative memory survived metamorphosis [117]. These findings raise questions about the mechanism of memory retention in metamorphosis - and the possibility of cellular memory storage. 

Our contemporary scientific understanding of memory is further challenged when we take a look at other living beings, such as plants. The possibility that plants could have memory is often disregarded due to their lack of a traditional brain or nervous system; yet they display an incredible capacity to remember, learn, and modify their behavior. One type of plant memory is “stress memory,” which occurs via “priming.” Priming is a process in which a brief exposure to stress “primes” the plant for subsequent stress by facilitating a more rapid and robust response of resistance. The two major mechanisms contributing to priming in plants are epigenetic and prion-mediated mechanisms [83]. 

Using the sensitive plant *Mimosa pudica*, animal ecologist Monica Gagliano described how a plant can learn habituation and then recall those memories in future situations to adapt its behavior [10]. *M. pudica* plants typically close their leaves when they are dropped. However, through repeated dropping experiments, some mimosa plants learned to ignore the stimulus of being dropped, reopening their leaves more quickly over successive trials. This form of learning persists even after a week, challenging traditional views on cognition and memory in plants. Gagliano also explored various potential explanations for memory storage in the absence of a brain, including calcium signaling, bioelectrical gradients, and epigenetic reprogramming.

Implications and future directions

This narrative review exploring cellular memory has several implications for future memory research. First, the studies referenced in this review indicate that a brain is not required to retain memories. Studies of single-cell organisms, such as bacteria, amoeba, and fungi, as well as multicellular organisms without brains, such as plants, demonstrate that memories can be retained and utilized by organisms to learn, even in the absence of a brain or a nervous system.

A second implication is that memory can be stored not only in the brain but also in cells outside the brain. Extra-cerebral memory has been demonstrated in the immune system, as well as in other cells in the body. Awareness of extra-synaptic memory may help explain reports of personality changes following organ transplants. Both anecdotal reports and clinical studies describe the transfer of memories from one individual to another via organ transplantation. This transfer of memory can result in personality changes in organ recipients who assume the traits of their donor. Furthermore, the psychological distress of those who have experienced such personality changes indicates the need for further investigation into the phenomenon of memory transfer following organ transplantation. Thus far, all studies exploring this phenomenon have been retrospective. Prospective studies are needed to confirm these changes. One possibility for structuring a prospective study would be to perform personality evaluations of living organ donors (e.g., kidney donors) and their recipients prior to surgery, then repeat these evaluations for the organ recipients postoperatively to determine if they acquired any new personality traits that are characteristic of their donor. These future studies could help improve the healing process of organ recipients and increase the psychological support for those undergoing transplant surgeries. 

Another potential implication of this study is the possibility that the same memory may be encoded and stored in different parts of the body. This would suggest that memories stored via synapses in the brain may also be stored in the peripheral nervous system, providing a backup system for memories. If replicated memories are found to exist, individuals who have lost memories, for example as a result of brain trauma or neurodegenerative disease, might be able to regain those memories if they were able to “download” these memories from organs outside the brain. These memories could be restored similar to the way certain organisms such as flatworms and metamorphosing butterflies are able to restore memories despite the loss or severe restructuring of their brains.

In summary, the findings of this review suggest that memory is not a singular function but rather a complex, multi-faceted operation that includes the encoding, storage, and retrieval of copious types of information in multiple locations, using innumerable mechanisms. This suggests that future research into the various processes involved in memory would benefit from a systems approach in which investigators specify the type of information being encoded, the method of encoding the information, the location where the information is stored, and the mechanisms by which this information is retrieved. This approach could help elucidate the specific processes involved in the particular type of memory being investigated.

Future research into the study of cellular memory has the potential to illuminate the pathophysiological mechanisms involved in memory impairment associated with various medical disorders, as well as brain trauma, while at the same time helping us gain a better understanding of extraordinary forms of memory, such as eidetic memory. Additionally, research into cellular memory may help explain how personality changes can occur following organ transplantation, foster the development of new therapeutic modalities to help individuals suffering from memory loss and assist individuals who do not suffer from any pathology in improving their memory.

## Conclusions

The exploration of cellular and non-neuronal memory systems presented in this review reveals a vast and largely uncharted domain that extends far beyond traditional models of cognition and memory. This narrative review highlights the finding that memories are stored both inside and outside the brain. Within the brain, memories are encoded and stored via synaptic changes, while outside the brain, subcellular components provide various mechanisms for storage. Anecdotal reports and clinical studies suggest some individuals may experience personality changes following organ transplantation, and these changes may involve the transfer of memories from donor to recipient. Prospective studies are needed to confirm or disprove this transfer of memories via organ transplantation. Understanding this phenomenon could inform the development of supportive therapies that enhance psychological outcomes for recipients, helping them process these changes and integrate their new experiences more effectively. As evidence mounts for memory storage in and out of the central nervous system, there is a pressing need to investigate how these mechanisms operate across different biological systems. Investigating these phenomena could transform our approach to understanding cognition and consciousness, leading to breakthroughs in medical treatments for neurological and psychological conditions. Interdisciplinary collaboration will be crucial in advancing these investigations, with the potential to yield innovative diagnostic tools and therapeutic strategies that significantly impact our understanding of memory.
